# NMDARs in Alzheimer’s Disease: Between Synaptic and Extrasynaptic Membranes

**DOI:** 10.3390/ijms251810220

**Published:** 2024-09-23

**Authors:** Sergio Escamilla, Javier Sáez-Valero, Inmaculada Cuchillo-Ibáñez

**Affiliations:** 1 Instituto de Neurociencias, Universidad Miguel Hernández-Consejo Superior de Investigaciones Científicas (UMH-CSIC), 03550 Sant Joan d’Alacant, Spain; j.saez@umh.es; 2 Centro de Investigación Biomédica en Red Enfermedades Neurodegenerativas (CIBERNED), 03550 Sant Joan d’Alacant, Spain; 3 Instituto de Investigación Sanitaria y Biomédica de Alicante (ISABIAL), 03010 Alicante, Spain

**Keywords:** NMDAR, GluN2B, GluN2A, GluN1, excitotoxicity, extrasynaptic NMDAR, Alzheimer’s disease

## Abstract

N-methyl-D-aspartate receptors (NMDARs) are glutamate receptors with key roles in synaptic communication and plasticity. The activation of synaptic NMDARs initiates plasticity and stimulates cell survival. In contrast, the activation of extrasynaptic NMDARs can promote cell death underlying a potential mechanism of neurodegeneration occurring in Alzheimer’s disease (AD). The distribution of synaptic versus extrasynaptic NMDARs has emerged as an important parameter contributing to neuronal dysfunction in neurodegenerative diseases including AD. Here, we review the concept of extrasynaptic NMDARs, as this population is present in numerous neuronal cell membranes but also in the membranes of various non-neuronal cells. Previous evidence regarding the membranal distribution of synaptic versus extrasynaptic NMDRs in relation to AD mice models and in the brains of AD patients will also be reviewed.

## 1. Structure, Function, and Subcellular Localization of NMDARs

N-methyl-D-aspartate receptors (NMDARs) are glutamate-binding calcium-gating channels involved in learning and memory processes [1,2,3]. NMDARs form tetrameric complexes assembled with two compulsory GluN1 subunits and two homomeric or heteromeric GluN2 (2A–2D) or GluN3 (3A–3B) subunits [4,5,6]. The four GluN2 subunits are major determinants of the heterogeneity of NMDAR function [4]. NMDARs are present in the whole central nervous system (CNS), with the highest densities in cortical and hippocampal structures [7,8]. The expression of NMDAR subunits, especially GluN2B, varies across different brain areas [9]. NMDAR density follows a gradient matching the cortical hierarchy, with neurons involved in more complex functions expressing more NMDARs [10]. The function of native NMDARs depends on their channel properties, abundance, and subcellular distribution between synaptic and extrasynaptic membranes [5,11]. This distribution defines their chemical micro-environment, its activation mode (tonic vs. phasic), and its interaction with different intracellular signaling molecules [12].

To fulfill their biological roles, most NMDARs are located at synaptic membranes, within the postsynaptic density (PSD) in neurons, being defined as synaptic NMDARs (SynNMDARs) [12]; however, NMDARs can also be located outside the synapses at a lower density than SynNMDARs [13], thus being defined as extrasynaptic NMDARs (ExsynNMDARs). This criterion usually includes those NMDARs in the perisynaptic space, such as the dendritic spine neck and places further from synapses in the dendritic shaft, the soma, or the axon [11,12,13,14]. Relying on morphological criteria, receptors are considered extrasynaptic when located at 100 nm or more from the PSD [12].

SynNMDARs and ExsynNMDARs display distinct roles in signaling pathways and gene regulation. SynNMDARs are important for LTP and prosurvival signaling [15]. Their activation produces phosphorylation and activation of the extracellular signal-regulated kinase (ERK) [16], phosphorylation of cAMP response element-binding protein (CREB) and neuroprotective effects [17]. On the other hand, the activation of ExsynNMDARs triggers the opposite mechanisms, as de novo long-term depression (LTD) [18,19], ERK dephosphorylation and inactivation, and shutting off of the CREB pathway. Pathological activation of ExsynNMDARs drives neuronal death through a process called excitotoxicity [17]. This process acts through mechanisms such as synapto-nuclear communication [20,21] and inversion of mitochondrial potential [22,23,24] and results in altered calcium influx [22,25,26].

In the adult human and mouse cortex, the most abundant subunits, along with GluN1, are GluN2A, GluN2B [4,27], and GluN3A, GluN3A being expressed more during the post-natal period [6,28]. GluN2A and GluN2B have different kinetics and biochemical properties [29,30] and different protein partners [31]. GluN2B is thought to be more mobile across membrane localizations than GluN2A [32]. Still, both GluN2A and GluN2B are present in synaptic and extrasynaptic membranes [13,33,34,35], with a complex and dynamic interplay between these two subcellular localizations. Furthermore, the presence of the GluN2A subunit increases NMDAR stability at synapses [15,22,32]. The consensus is that GluN2A and GluN2B are mainly synaptic [34], while GluN3A is mainly associated with the perisynaptic site of the PSD [28,36]. Remarkably, extrasynaptic GluN2A and GluN2B are related to excitotoxicity [35,37,38,39]. Therefore, changes in the distribution of NMDAR subunits can affect synaptic stability and play a role in various neurodegenerative diseases [40].

## 2. How to Distinguish SynNMDARs and ExsynNMDARs

Approaches to analyzing the synaptic/extrasynaptic distribution of NMDARs are based on imaging analysis, electrophysiological studies using pharmacologic tools, and biochemical fractionation. Imaging tools such as electron micrographs and confocal or high-resolution microscopy identify SynNMDARs when they colocalize with a protein present in the PSD, typically PSD95 [41,42], or with the presynaptic proteins synaptophysin or synapsin 1 [43,44]. Specific pharmacological drugs distinguish synaptic and extrasynaptic NMDARs based on their capacity to block preferentially one over the other. For instance, MK-801 blocks SynNMDARs preferentially [17,45], while memantine blocks ExsynNMDARs preferentially [46,47]. Other drugs act on specific subunits, such as ifenprodil, that block GluN2B preferentially [44,48], and this is useful in electrophysiological characterization.

Biochemical fractionation protocols can isolate SynNMDARs and ExsynNMDARs based on the differential solubility of the plasma membranes where they are located. The PSD-containing membranes are very dense and contain a meshwork of proteins linking synaptic receptors to signaling molecules and the cytoskeleton [49]. Consequently, these membranes are insoluble in solutions with low detergent concentrations and generate a pellet after centrifugation, mainly composed of the PSD, and thus, it is considered the synaptic fraction. Conversely, those plasmatic membranes not attached to the PSD are highly soluble in detergent solutions and remain in the supernatant after centrifugation, representing the extrasynaptic fraction [50,51]. Different biochemical fractionation protocols exist for PSD isolation [52,53,54,55], mainly designed and tested for fresh mice brains.

### The Conception of ExsynNMDARs

**Figure 1:**
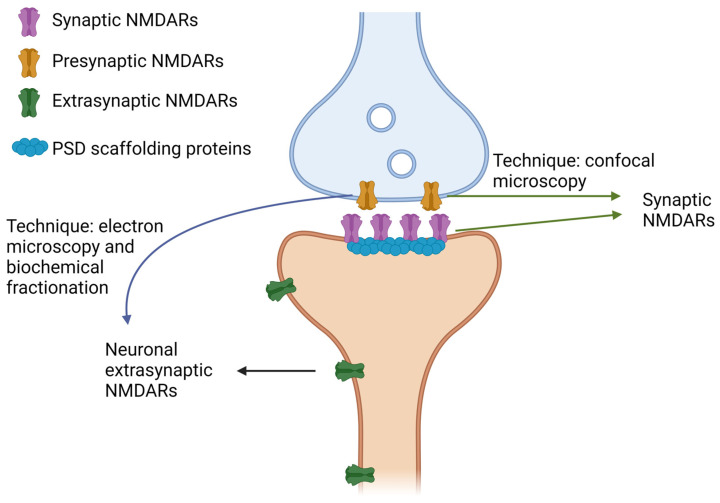
Classification of neuronal NMDARs as synaptic or extrasynaptic according to the technique of choice. Schematic illustration of a glutamatergic synapse, including the pre (blue)- and postsynaptic (orange) terminals. Different populations of NMDARs are represented: (1) presynaptic, (2) those located in the PSD, and (3) extrasynaptic. Synaptic NMDARs include those in the PSD and the presynaptic ones when the technique of choice is confocal microscopy, especially when the synaptic marker is a presynaptic protein, such as synaptophysin and syntaxin 1. However, when the technique is biochemical fractionation, presynaptic NMDARs will reside in the extrasynaptic fraction, and the synaptic fraction will be composed mainly of the PSD. In addition, electron microscopy allows us to distinguish pre- from postsynaptic terminals and, thus, presynaptic NMDARs and those in the PSD. Created in BioRender.com.

SynNMDARs are primarily found in the postsynaptic membranes of glutamatergic excitatory neurons. However, they have also been identified in inhibitory GABAergic interneurons in mice [56,57,58,59,60]. In contrast, the term “ExsynNMDARs” is ambiguous and not well established. Typically, ExsynNMDARs refer to neuronal NMDARs located in the plasma membrane outside the PSD, dendritic shaft, and soma. This category may also encompass presynaptic NMDARs, which have distinct synaptic transmission and plasticity functions, although their function is less explored [14,59,61]. This raises concerns about grouping specific NMDARs located within and outside of synapses under the blanket term of ExsynNMDARs (Figure 1).

Overall, neuronal ExsynNMDARs may have specific functions that differ from synaptic NMDARs. ExsynNMDARs may be in extrasynaptic membranes because they are in transit, either being stored temporarily or actively moving to synapses from exocytosis sites or synapses to sites of endocytosis [62,63]. However, they could reside permanently in extrasynaptic membranes organized in supramolecular structures like their synaptic counterparts. Most of these extrasynaptic sites are points of contact with adjacent processes, including glia, axons, synaptic terminals, and dendrites [13,64].

Furthermore, it is important to note that ExsynNMDARs may also refer to non-neuronal NMDARs, expressed by astrocytes [65,66,67], microglia [68,69,70], oligodendrocytes [71], and endothelial cells [72,73].

In immunofluorescence studies, “synaptic NMDARs” refer to the population of NMDARs in the PSD that typically colocalizes with PSD95 [41,74,75]. However, other postsynaptic markers such as Homer [28,76,77,78,79] or Shank [77] are also used. Another typical criterion for defining SynNMDARs is the colocalization with a presynaptic marker, usually synaptophysin [43,44], which would include presynaptic NMDARs as SynNMDARs. To standardize the protocol for measuring synaptic and extrasynaptic NMDARs, the best approach to identify SynNMDARs would likely be to use a combination of pre- and postsynaptic markers [34,80], since both pre- and postsynaptic terminals are needed to build a synapse.

It is not always clear whether ExsynNMDARs are free or part of protein complexes. Some candidates associated with neuronal ExsynNMDARs are protein phosphatase 1 (PP1) [74], adhesion proteins such as cadherin and catenin [13], the C-terminus of GIPC (G α-interacting protein) [81], or membrane-associated guanylate kinases (MAGUKs) [11] such as SAP102 [62,82] or SAP97 [83]. These proteins may not be exclusively confined to a single membrane compartment (synaptic or extrasynaptic), making it challenging to distinguish between synaptic and extrasynaptic NMDARs [13,82,83]. In this line, PSD95, essentially postsynaptic, was found by immunofluorescence and electron-microscopy immunogold images in extrasynaptic membranes in clusters containing NMDARs [13]. This suggests that neuronal NMDARs attached to PSD95 could not be considered exclusively as SynNMDARs, and some overestimation of this population could occur when using imaging techniques.

**Figure 2:**
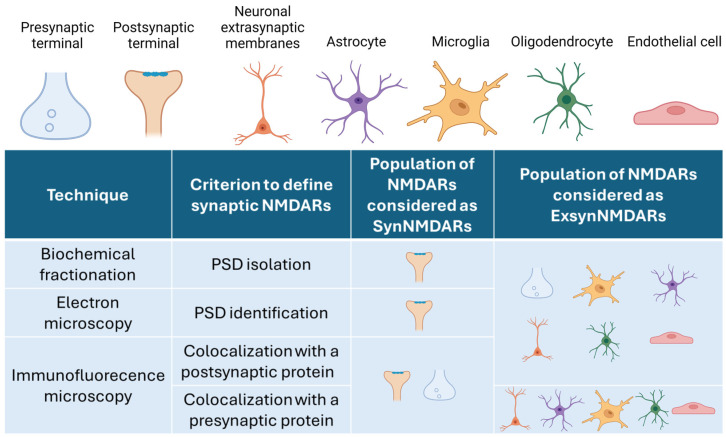
Classification of NMDARs as synaptic or extrasynaptic according to the technique of choice and cell type. The schematic table contains columns for the technique of choice, the criterion to define an NMDAR as synaptic, and which NMDAR populations will be considered as SynNMDARs or ExsynNMDARs attending to subcellular localization or cell type origin. Created in BioRender.com.

When immunofluorescence is the technique of choice, the type of biological sample determines the necessary precautions to prevent mixing NMDARs from different cell types. In pure neuronal cultures, neuronal ExsynNMDARs will be those that do not colocalize with synaptic markers since there are no other cell types. However, in cultures containing non-neuronal cells (e.g., mixed neuronal and astrocytic cultures), brain tissue slices, or brain organoids, ExsynNMDARs will correspond to different populations. NMDARs that do not colocalize with synaptic markers but do with neuron-specific cytoskeletal markers, such as class III beta-tubulin (TUJ1) or MAP2, will correspond to neuronal ExsynNMDARs, whereas NMDARs that colocalize with markers, such as GFAP or S100β (astrocytes) or iba1 (microglia), will correspond to non-neuronal ExsynNMDARs (astrocytic and microglial NMDARs, respectively) (Figure 2). When biochemical fractionation is the technique of choice and a piece of brain is the starting material, the extrasynaptic fraction will contain NMDARs from different cell types besides neurons, such as astrocytes, microglia, oligodendrocytes, and endothelial cells [84].

Finally, specific blockers such as MK-801 and memantine are used to discriminate the activity of neuronal SynNMDARs and ExsynNMDARs, but these drugs also block ExsynNMDARs from astrocytes [65,85,86] and microglia [68,69,86,87], highlighting the need for precise characterization of the ExsynNMDAR populations. Electron-microscopy images can discriminate between presynaptic and postsynaptic NMDARs. This technique has shown the presence of presynaptic NMDARs at rat cortical presynaptic terminals, where immunostaining was sparse and substantially less intense than postsynaptic staining [61]. Cellular fractionation is another tool for isolating presynaptic from postsynaptic NMDARs [84].

When the sample includes different cell types, such as those in brain slices, cerebral organoids, and in vitro co-cultures, it is important to consider that NMDARs are expressed not only by neurons but also by astrocytes, microglia, oligodendrocytes, and endothelial cells. Depending on the technique of choice, the NMDARs considered synaptic or extrasynaptic will differ. Biochemical fractionation will isolate the PSD. Thus, SynNMDARs will be those in the PSD, and the ExsynNMDARs will be the rest. Electron microscopy allows the identification of the PSD. Thus, it will be able to consider the NMDARs in the PSD, the presynaptic and the extrasynaptic NMDARs independently. Immunofluorescence-microscopy criteria rely on the colocalization of NMDARs with either synaptic or extrasynaptic proteins. The most used postsynaptic marker is PSD95, which is considered to reside exclusively in the PSD (even though it has been argued that PSD95 could also be present in extrasynaptic membranes [13]). However, the pre- and postsynaptic terminals are so close to each other that they will colocalize, meaning that immunostaining from presynaptic NMDARs and those NMDARs in the PSD will be mixed, being both populations will be considered as SynNMDARs. When the choice is a presynaptic marker (usually synaptophysin or syntaxin1), the result will be similar, since NMDARs will colocalize with those in the PSD and presynaptic NMDARs.

## 3. NMDAR Distribution in Alzheimer’s Disease

It is assumed that an imbalance between SynNMDAR and ExsynNMDAR activation could be part of the etiology of neurodegenerative diseases such as AD [36,88,89,90], where the homeostasis of glutamate is dysregulated [91,92,93]. However, there is relatively little information about alterations in the distribution of NMDARs in synaptic and extrasynaptic membranes in the brains of individuals with AD. One of the few drugs used in AD therapy, memantine, is an open-channel blocker of ExsynNMDARs [46,47,94,95]. Memantine is currently used in combination with acetylcholinesterase inhibitors [96], and despite the clinical effects being controversial still [97], the data in preclinical studies suggest that it has a positive impact on improving AD brain neuropathology [98].

Chronic activation of ExsynNMDARs could be a contributing effector of AD [36,99,100,101]. In vitro and in vivo studies suggest an excessive release of glutamate from astrocytes in AD activates ExsynNMDARs in neurons [102]. Moreover, the activation of ExsynNMDARs increases the production of the β-amyloid peptide (Aβ) [103] and increases the expression [41,104,105] and phosphorylation [102] of tau, the main hallmarks of AD. In this context, it has been reported that pharmacological inhibition of GluN2B ameliorates tau pathology [104,105,106]. On the contrary, stimulation of SynNMDARs increases the non-amyloidogenic processing of APP by α-secretase, thus decreasing the release of Aβ [107].

AD is usually modeled in vitro and in vivo using transgenic mice over-expressing human APP or by adding Aβ peptides [41,43,108], but tau pathology can also be modeled [109,110]. Tau is a cytoskeleton protein mainly present in the axon but also in the dendritic compartment [111]. Several studies show a relation between tau and NMDARs through the stabilization of NMDARs at the PSD [112] and, more specifically, regulating ExsynNMDAR lateral diffusion. However, the possible alteration in the NMDAR distribution in tau models of AD has not been fully explored. We will independently review the impact of these two pathological mechanisms on the distribution of SynNMDARs and ExsynNMDARs.

### 3.1. Distribution of SynNMDARs and ExsynNMDARs in Animal Models of AD

#### 3.1.1. Distribution of SynNMDARs and ExsynNMDARs in Tauopathy Mice Models

Levels of ExsynNMDAR subunits have been analyzed in the AD mice model expressing P301S, a human mutant tau that leads to the widespread neurofibrillary tangles of phospho-tau, resembling the neurofibrillary tangles found in the brains of patients with AD. In these mice, the subcellular localization of GluN1 has been analyzed using electron micrographs of the hippocampus [113]. In this study, synaptic GluN1 in excitatory synapses and interneuron dendrites was significantly reduced in P301S mice, while extrasynaptic GluN1 increased in interneuron dendrites, with respect to wild-type mice. This differential distribution of synaptic versus extrasynaptic NMDARs supports the notion that the progressive accumulation of phospho-tau is associated with changes in NMDAR distribution since these alterations are observed at 10 months old when pathology is present, but not at 3 months old. In agreement, our recent analysis of NMDAR subunit distribution in this AD model, using a subcellular fractionation protocol, also resulted in lower levels of synaptic GluN1 and GluN2B and also lower levels of extrasynaptic GluN3A, with respect to those in wild-type mice [84].

In another model of tauopathy, the rTg4510 mouse, which also expresses P301L human tau associated with FTDP-17 [114], the authors of a study reported that human tau and mutant P301L tau are enriched in dendritic spines of rTg4510 compared to control mice. In parallel, the synaptic expression of GluN1 and GluN2/3 was lower in rTgP301L mice.

These studies with tau mice models indicate that tau phosphorylation can play a role in NMDAR distribution, probably through tau mislocalization to dendritic spines, rich in F-actin [115], and lead to an impaired intracellular sorting and trafficking of synaptic proteins [116], including NMDARs.

Accordingly, it has been hypothesized that tau hyperphosphorylation could lead to increased levels of NMDARs in the extrasynaptic membranes. In a recent study, researchers reached these conclusions by using *crmp1* KO mice [117]. CRMP1 is a protein that regulates F-actin depolymerization and is associated with synaptic plasticity mechanisms [118,119]. To identify NMDAR distribution, they used a fractionation protocol with PSD95 as a synaptic marker. They found in the *crmp1* KO mice increased ExsynNMDAR subunit levels, accompanied by increased levels of phosphorylated tau, and claimed that CRPM1 and tau malfunction could lead to F-actin depolymerization in the dendritic spine and concomitant increase in ExsynNMDARs.

The effect of tau on NMDAR distribution was also tested in tau-KO mice [120]. The authors of a study analyzed, by immunohistochemistry, the association of GluN2B-Y1336 phosphorylation (phosphorylation that has been associated mainly with extrasynaptic localization [121]) with extrasynaptic GluN2B subunits. They observed that the absence of tau leads to a decrease in functional ExsynNMDARs in the hippocampus and proposed that tau is involved in NMDAR trafficking through actin depolymerization in the spine [122] as a possible mechanism that regulates NMDAR lateral diffusion.

In the same line of evidence, in mice primary hippocampal neurons treated with tau derived from the brains of patients with AD, GluN2B was translocated from the synapse to extrasynaptic membranes, identified by imaging colocalization with PSD95 or by biochemical fractionation [41]. Authors pointed out that, in these cultures, tau derived from AD was able to increase Casein Kinase 2 (CK2), which phosphorylates GluN2B in serine 1480, detaching this subunit from PSD95. This enhances the probability of GluN2B of leaving the synapse by either lateral diffusion or by endocytosis [75,80]. Interestingly, the levels of CK2 are increased in the hippocampus of patients with AD [123] but not in other tauopathies.

Together, these data indicate that the tauopathy that develops in the brains of individuals with AD could promote the translocation of NMDAR subunits from the synaptic to the extrasynaptic membranes.

#### 3.1.2. Distribution of SynNMDARs and ExsynNMDARs in Aβ-Treated Cultures and Mice Models

Aβ is related to spine loss by reducing SynNMDAR levels [124]. A pioneering study in cultured cortical neurons showed that Aβ enhances the activity of the phosphatase STEP61, which dephosphorylates GluN2B at Tyr1472, inducing its endocytosis through clathrin adaptor proteins [43], while extrasynaptic and total NMDARs levels remained unchanged. In agreement, in mice hippocampal slices, a combination of current blockage by MK-801, biochemical fractionation, and confocal colocalization with synapsin determined that prolonged exposure to soluble Aβ oligomers (hours), but not brief exposure (minutes), decreases synaptic GluN2B while extrasynaptic GluN2B remains unaffected [44].

Most of the in vitro studies that evaluate Aβ effects on NMDAR levels in murine hippocampal or cortical cultures do not discriminate between SynNMDARs and ExsynNMDARs and, instead, evaluate NMDAR total levels or the surface expression of NMDAR subunits. These studies describe that Aβ reduces the surface expression of GluN1 and GluN2B [48,53,125,126], although the total levels do not change, and causes a reduction in the number of GluN2A-positive dendritic spines [127]. Similarly, in rat entorhinal cortex slices, 3 h of exposure to Aβ decreases GluN2B and GluN2A total protein levels and GluN2B mRNA levels, but no changes were observed in GluN1 [128].

The discrepancy between the results obtained regarding NMDAR subunit levels when reported as being associated with membranes and those of the total levels could be explained by the population of NMDARs residing in intracellular pools. In cerebellar granule cells, the majority of unassembled GluN1 subunits are located in the endoplasmic reticulum [129]. This could mask possible reductions in GluN1 in synaptic and extrasynaptic membranes precisely when levels are measured in total cell extracts without any fractionation protocol to distinguish them or in immunofluorescence assays in permeabilization conditions.

Other studies have also evaluated NMDAR levels in the brain of the APP/PS1 AD mice model [109,110], which develops amyloid plaques and shows AD-like cognitive impairment. Reduced levels of GluN2B alone or with GluN1 have been observed in these models in the synaptic fraction obtained by biochemical fractionation of the hippocampus [53,126]. Indeed, when a fractionation protocol is employed to isolate synaptic and extrasynaptic membranes, low levels of synaptic GluN2B and high levels of extrasynaptic GluN2B have been described in the hippocampus of these AD mice [52]. In our recent study, we observed low levels of GluN1 in synaptic and extrasynaptic membranes in the cortices of APP/PS1 mice [84], which are likely affecting all NMDARs and, therefore, contributing to the synaptic failure described in this model [130] driven by Aβ.

### 3.2. NMDAR Subunit Levels in the Brain of Individuals with AD

Firstly, it is essential to note that the methodological approaches to studying the NMDARs in the human post-mortem brain are hindered by preanalytical confounding factors, such as freeze/thaw cycles [131] and the post-mortem intervals (PMI) of the samples. It is well established that NMDAR subunits are vulnerable to PMI-associated degradation in different degrees. Indeed, the GluN1 subunit protein is unaffected by post-mortem delays up to 18 h, while GluN2A and GluN2B subunit proteins show significant degradation with shortened PMI [132,133].

Currently, brain banks aim to reduce PMI to just a few hours. However, overall rRNA and mRNA stability are maintained for up to 60 h post-mortem [131,134], without apparent correlation with pH changes due to tissue acidification [34], although specific mRNAs may be selectively degraded [35]. Synaptosomes isolated from frozen human brain retain respiratory activity and the ability to release neurotransmitters and appear to be morphologically indistinguishable from those from fresh tissues, even with a PMI of 24 h [135]. On the other hand, dephosphorylation may occur on some proteins in less than 1 min, which is a significant problem even in animal experiments [36].

Ideally, the effect of PMI should be individually addressed for each assay condition, but this may not be practical in many experiments. To address degradations, protocols for estimating NMDAR degradation have been proposed [133,136] to allow researchers to discard brain samples with high synaptic degradation [132]. For example, the HUman Synapse Proteome Integrity Ratio or “HUSPIR index” aims to evaluate the integrity and preservation of the post-mortem samples prior to analyses, and to obtain this, this index measures the ratio of two proteolytic fragments of GluN2B in synaptic fractions by immunoblots [136].

Studies of NMDAR expression in human samples are few in comparison with those in mice models. In the human cortex, the evaluation of NMDAR levels has been approached by transcriptional techniques and by measuring total protein levels from brain extracts without the capacity to distinguish SynNMDARs from ExsynNMDARs. Techniques that allow us to distinguish them, such as subcellular fractionation, are quite scarce.

#### 3.2.1. Regional NMDAR Transcript Levels in the Brain of Individuals with AD

Studies that have evaluated NMDAR subunit expression using RT-qPCR report reduced mRNA levels of GluN1, GluN2A, and GluN2B in the hippocampus, temporal cortex, entorhinal cortex, and cingulate cortex from individuals with AD and report no alterations in less vulnerable regions, such as the occipital cortex or cerebellum [132,137,138,139]. Novel transcriptomic technologies, such as single-cell transcriptomics, have focused the analysis on the expression of *GRIN1*, the gene that codifies the compulsory NMDAR subunit GluN1. *GRIN1* is downregulated in the temporal cortex of individuals with AD [134,140]. In the prefrontal cortex, *GRIN1* expression is modulated through AD progression, being upregulated at the beginning of the disease, but is eventually downregulated with respect to controls [141]. Other studies do not find any change in the expression of *GRIN1* in the frontal or prefrontal cortex [134,142] nor when *GRIN1* was assessed in astrocytes [143]. Transcriptomic expressions of other NMDAR subunits, *GRIN2A*, *GRIN2B*, and *GRIN3A*, are downregulated in the temporal cortex of individuals with AD [140].

#### 3.2.2. Total Protein Levels of NMDAR Subunits in the Brain of Patients with AD

The expression of NMDAR subunits at the protein level measured by immunoblots closely follows the expression at the transcript level. Accordingly, levels of GluN1, GluN2B, and GluN2A are reduced in extracts from AD-susceptible regions such as the hippocampus, entorhinal cortex, frontal cortex, or cingulate cortex from individuals with AD with respect to controls [132,133,138,144], but no changes are reported in less susceptible regions, such as the occipital cortex or the caudate [144]. However, some studies have found increased levels of GluN2A in the hippocampus at moderate stages of AD [132], and increased GluN2B levels in the prefrontal cortex at the earliest stages of the disease [145]. The employment of quantitative in vitro autoradiography with the specific NMDAR antagonist [^3^H]MK-801 [146], which allows the quantification of global levels of NMDARs, also shows lower levels of the receptor in the hippocampus and entorhinal cortex but not in the basal ganglia in individuals with AD.

In summary, most of the previous reports concluded that total protein and transcript levels of NMDAR subunits decrease in susceptible brain areas in AD. Interestingly, high levels of GluN1 and GluN2A were recently described [147] using confocal microscopy in the astrocytes of the hippocampus of individuals with AD (Braak stage IV–VI) but not in neurons.

This result highlights that the levels of NMDARs could change in the AD brain in different compartments of neurons and other cell types. In this regard, little is known about what functions NMDARs perform in non-neuronal cells (reviewed here for astrocytes [67,148], oligodendrocytes [149,150], microglia [70,151], and non-neuronal cells in general [152], respectively). Overall, this suggests that changes in the levels of NMDARs from different populations are likely contributing to different manifestations associated with AD progression.

#### 3.2.3. NMDAR Subunits Protein Levels in Synaptic and Extrasynaptic Membranes

Studies performed in animal models and primary cell cultures led to the idea that GluN2A populates mainly the synaptic membranes, while GluN2B is mostly extrasynaptic [5,32,37,153]. Thus, the activation of GluN2A would lead to LTP and prosurvival signaling, while GluN2B would be responsible for LTD and excitotoxicity [154]. However, this oversimplified model was rapidly challenged by two main experimental outcomes. First, both GluN2A and GluN2B subunits populate synaptic and extrasynaptic membranes [34]. And second, both subunits participate in excitotoxicity [35,155].

Overall, these results may vary due to differences in experimental conditions. The use of different neurodevelopmental stages and the absence of pharmacological tools to definitively distinguish NMDAR subtypes may account for the conflicting outcomes [88]. The “age” of cultured neurons is another critical factor. After one week of culture, around 90% of NMDARs are in the extrasynaptic membranes, while this number reduces to 50% or less after two weeks in vitro [11]. These conflicting results strengthen the need for studies performed on the human brain.

**Figure 3:**
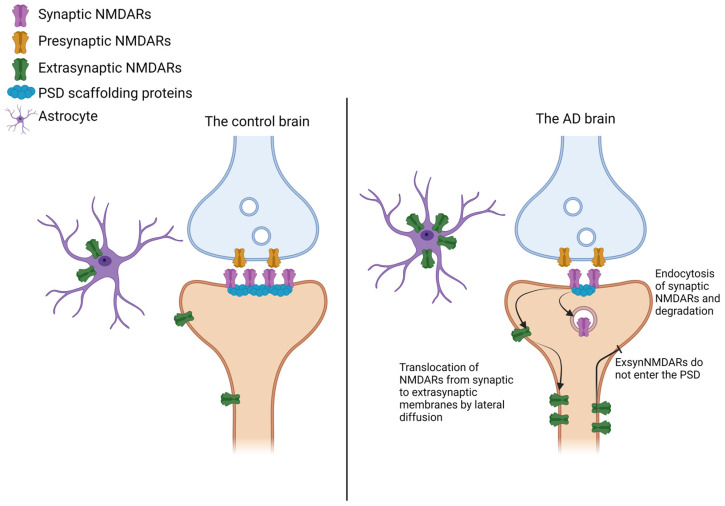
Model of altered levels of NMDARs in the AD brain.

In this regard, subcellular fractionation methods permit the isolation, purification, and/or enrichment of specific cellular compartments from complex tissue samples [156,157,158,159,160] that allow unique insights, resulting in them being more informative than the assessment of total protein levels. In a recent study, we optimized the fractionation protocol of post-mortem human brain cortex [84], allowing us to describe for the first time the distribution of the main four NMDAR subunits—GluN2B, GluN2A, GluN1, and GluN3A—between synaptic and extrasynaptic membranes in the human frontal cortex. An analysis of the total levels of NMDAR subunits on crude membrane fractions from AD cortex displayed, in good agreement with previous studies, decreased levels of GluN1, GluN2B, and GluN2A, with unchanged GluN3A levels, with respect to controls. Our analysis of the synaptic membranes demonstrated that GluN2B and GluN2A levels were lower in AD than in controls. More interestingly, when we quantified the extrasynaptic membrane levels of GluN2B and GluN1, these were higher in AD, and GluN2A showed a similar trend. Remarkably, we found two different glycoforms of GluN2B and GluN2A in the extrasynaptic membrane that turned out to be increased in an AD brain. Our study uncovered the NMDAR distribution in an AD cortex, showing a reduction in NMDARs in synaptic membranes and an increase in extrasynaptic membranes. The shift to extrasynaptic membranes of GluN2B, GluN2A, and GluN1 reported could explain the exacerbated NMDAR-related excitotoxicity observed in AD (Figure 3).

Several studies suggest that SynNMDARs are lower in the AD brain while ExsynNMDARs are increased. Possible explanations for the decrease in SynNMDARs include endocytosis and posterior degradation or lateral diffusion. The increase in ExsynNMDARs can be explained by the translocation of NMDARs from the PSD to extrasynaptic membranes, impaired delivery of NMDARs to the PSD, and increased expression of NMDARs by non-neuronal cell types, such as astrocytes. Created in BioRender.com.

## 4. Conclusions

The distribution of synaptic versus extrasynaptic NMDARs has emerged as an important parameter that contributes to neuronal dysfunction in neurodegenerative diseases such as AD [11,88]. Protein hallmarks of AD pathology, tau, and beta-amyloid peptide contribute to the imbalance by promoting SynNMDAR endocytosis [43,44] and increasing ExsynNMDARs [52]. Overall, studies in AD mice models and in the human brain from individuals with AD indicate that SynNMDAR levels are reduced while ExsynNMDAR levels increase with respect to controls (Table 1 and Table 2). Whereas the activation of SynNMDARs is neuroprotective [17,22], the activation of ExsynNMDARs has neurotoxic effects linked to neuronal death. Consequently, any alteration in the number and density of NMDARs could contribute to the synaptic and memory deficits that are associated with AD. Consequently, distinguishing synaptic from extrasynaptic NMDARs is particularly important for defining therapeutic approaches.

ExsynNMDARs include a broader population of receptors than those included in the term SynNMDAR. Proper criteria are necessary to characterize ExsynNMDARs since neuronal and non-neuronal cells express ExsynNMDARs, and an imprecise identification can arise if it is assumed that most of the ExsynNMDARs are exclusively neuronal. Subcellular fractionation protocols allow us to isolate NMDARs from the PSD (synaptic fraction) from those outside the PSD (extrasynaptic fraction). While the NMDARs in the synaptic fraction are well defined, the NMDARs in the extrasynaptic fraction are a mix of presynaptic, neuronal extrasynaptic, and non-neuronal. However, no further assessments are usually performed to gain insight in this regard. Furthermore, a technique as common as immunofluorescence in neuronal cultures can identify “synaptic NMDARs” without discriminating those located in the post- and presynaptic membranes unless higher-resolution techniques are utilized [158], such as 3D reconstructions of isolated spines [61]. Therefore, a correct identification of ExsynNMDARs is necessary since their role is not yet fully understood.

In the clinic, NMDARs are currently the targets of numerous programs for finding new drugs for AD or other diseases of the CNS [161,162]. The correct discrimination among all the types of NMDARs present in the brain will benefit the research for specific drugs, to help cure these diseases.

## Figures and Tables

**Table 1:** Summary of studies assessing synaptic and extrasynaptic NMDAR subunit protein and mRNA levels in human models. N/A: non-applicable.

**mRNA Levels**								
**Reference**	**Year**	**Technique**	**Brain Area**	**Sample Size (AD Braak Stage)**	**Levels with Respect to Control**			**Cell Type** **(When Specified)**
					***GRIN1***	***GRIN2A***	***GRIN2B***	
[139]	2001	qPCR	Temporal and cingulate cortex	10 (no Braak specified)	Down	N/A	N/A	
[138]	2004	qPCR	Hippocampus, anterior cingulate gyrus, and superior temporal cortex	10 (no Braak specified)	Down	Down	Down	
[132]	2004	qPCR	Hippocampus	10 (I–II); 10 (III–IV); 10 (V–VI)	Down	No change	Down	
[137]	2002	qPCR	Hippocampus	10 (no Braak specified)		Down	Down	
[142]	2010	Microarray	Prefrontal cortex	14 (I–II); 14 (III–IV); 14 (V–VI)		Down		
[141]	2019	snRNAseq	Prefrontal cortex	10 (I–II); 21 (III–IV); 17 (V–VI)	Up at early stages but down at late stages	Down	No change	Excitatory neurons
[140]	2024	RNAseq	Superior temporal gyrus	10 (V–VI)	No change	No change	No change	
[134]	2020	RNAseq	Prefrontal cortex	12 (IV–VI)		Up	Up	Endothelial cells
					Down	Down		Oligodendrocytes
**Total Protein Levels**								
**Reference**	**Year**	**Technique**	**Brain Area**	**Sample Size (AD Braak Stage)**	**Levels with Respect to Control**			**Cell Type (When Specified)**
					**GluN1**	**GluN2A**	**GluN2B**	
[138]	2004	WB	Hippocampus, anterior cingulate gyrus, and superior temporal cortex	10 (no Braak specified)		Down	Down	
[132]	2004	WB	Hippocampus		Down	Up (in early stage)	Down	
[146]	2013	Quantitative autoradiography	Hippocampus	23 (IV–VI)	General NMDAR reduction	General NMDAR reduction	General NMDAR reduction	
[144]	2001	WB	Entorhinal cx	6 (III–VI)	No change	Down	Down	
			Hippocampus		Down	No change	Down	
			Caudate		No change	No change	No change	
			Occipital cortex		No change	No change	No change	
[147]	2021	Quantitative confocal microscopy	Hippocampus	8 (IV–VI)	Up	Up		General and specifically in astrocytes
[133]	2000	WB	Hippocampus	6 (no Braak specified)	Down	No change	Down	
			Frontal cx		Down	Down	Down	
			Entorhinal cx		No change	No change	No change	

**Table 2:** Summary of studies assessing synaptic and extrasynaptic NMDAR subunit protein and mRNA levels in mice AD models. An asterisk means an additional explanation in the ‘Other findings’ column.

**Tauopathy Mice Models**										
**Reference**	**Year**	**Technique**	**Criterion SynNMDAR**	**Criterion ExsynNMDAR**	**Model/Cell Culture Treatment**	**NMDARs Levels Respect to WT or Control**			**Observations**	**Other Findings**
						**SynNMDAR**	**ExsynNMDAR**	**Total NMDAR**		
[120]	2019	Microscopy	Y1472-GluN3B	Y1336-GluN3B	tau KO mice	No change	No change	No change	Hippocampus	tau KO lacks ExsynNMDAR currents
[112]	2010	Biochemical	Solubility in SDS	Solubility in pH 8	tau KO mice	Down	Up	No change	Hippocampus	
[113]	2023	SDS-FRL	(Self-developed semi-automatic software) Dendritic spines were considered as such if (1) they emerged from a dendritic shaft or (2) they opposed an axon terminal recognized by the presence of synaptic vesicles on their cross-fractured portions	Non-specific background labeling was measured on E-face structures surrounding the measured P-faces (specific staining surrounding spines)	Tg P301S mice	No change *	Up **		* In excitatory neurons, decreased SynNMDARs but unaltered ExsynGluN1	** Specifically in interneuron dendrites of the stratum oriens
[41]	2022	Microscopy	Colocalization with PSD95	The rest	Neurons treated with tau from AD brain tau for 7 days	Down	Up	Down	Mouse cultured hippocampal neurons	
**Amyloidosis Mice Models**										
**Reference**	**Year**	**Technique**	**Criterion Syn NMDAR**	**Criterion ExsynNMDAR**	**Treatment/Model**	**NMDARs Level Respect to WT or Control**			**Observations**	**Other Findings**
						**SynNMDAR**	**ExsynNMDAR**	**Total NMDAR**		
[43]	2005	Microscopy	Colocalization with synapsin	No colocalization with synapsin	Cultured cortical neurons treated with Aβ 1 h	Down GluN1	Suggests redistribution to extrasynaptic membranes			Detect reduced GluN1 in surface levels but no changes in total levels. Suggests redistribution to extrasynaptic membranes.
		Biotinylation						No change	Reduced surface expression of GluN2B and GluN1, no change in total levels	
[44]	2011	Biochemical	Triton soluble fraction	Triton insoluble fraction	Mice slices treated with Aβ -> fractionation	Down GluN2B	No change			
		Microscopy	Colocalization with synapsin	No colocalization with synapsin	Cultured hippocampal neurons + Aβ	Down GluN2B	No change			
[52]	2023	Biochemical	Triton insolubility	Triton solubility	APP/PS1 mouse	Down GluN2B	Up GluN2B			
